# Culture-dependent and -independent investigations of bacterial migration into *doenjang* from its components *meju* and solar salt

**DOI:** 10.1371/journal.pone.0239971

**Published:** 2020-10-13

**Authors:** Jung-Min Lee, Sojeong Heo, Yoon-Su Kim, Jong-Hoon Lee, Do-Won Jeong

**Affiliations:** 1 Department of Food and Nutrition, Dongduk Women’s University, Seoul, Republic of Korea; 2 Department of Food Science and Biotechnology, Kyonggi University, Suwon, Republic of Korea; Kyungpook National University, REPUBLIC OF KOREA

## Abstract

We determined bacterial migration into *doenjang* from its components, *meju* and solar salt using culture-based and 16S rRNA gene-based culture-independent techniques (pyrosequencing of total DNA). Pyrosequencing results suggested that the bacterial communities of *meju*, but not solar salt, significantly affected those of *doenjang* communities. Culture-based pyrosequencing analysis yielded similar results. These results indicate that most predominant bacterial species in *doenjang* migrated from *meju*, not solar salt. We therefore believe that the present study is one of the most comprehensive comparisons of bacterial communities of fermented soybeans using culture-dependent and -independent methods. Furthermore, pyrosequencing of the V3 and V4 regions of bacterial 16S rRNA did not distinguish among *Bacillus amyloliquefaciens*, *B*. *siamensis*, and *B*. *velezensis* as well as between *Enterococcus faecium* and *E*. *hirae*.

## Introduction

*Doenjang* is a traditional Korean high-salt-fermented soybean paste used as a sauce, ingredient, or both for a variety of foods because of its nutritional and sensory properties [1]. The microbial communities of *doenjang* confer its quality and flavor [2]. Culture-dependent results suggested that most bacteria involved in *doenjang* fermentation are derived from raw materials such as its major component *meju* (fermented soybean block), which is prepared from soybeans by soaking, steaming, mashing, and molding, followed by fermentation for one or two months under natural environmental conditions [1]. *Doenjang* prepared from ripened meju contains approximately 18% high-salt brine [3,4]. The salt contents of *meju* and *doenjang* are significantly different (>10% w/v), and Jeong et al [5] suggested that difference of salts affects the compositions of microbial communities.

Recently, culture-independent methods such as pyrosequencing, which are increasingly applied to determine the composition of microbial communities in fermented soybean, have identified diverse microorganisms, including previously unidentified species that are not detected using culture-dependent methods [6–10]. However, the migration of the microbial community from *meju* to *doenjang* remains uncharacterized because it is difficult to obtain the same lots of *meju* because of the long fermentation process (>4 months) [5].

In our previous culture-dependent study to investigate bacterial migration from *meju* to *doenjang*, we identified *Enterococcus* and *Tetragenococcus* as the dominant genera in *meju* and *doenjang* from the Gyeonggi Province of Korea, respectively; and *Bacillus* is the dominant genus throughout the entire process [5]. These results indicate a shift in the salt concentration of *meju* (approximately 1.5%) to *doenjang* (approximately 12%) that is mediated by the dominant species. The predominance of salt-tolerant bacteria, including *T*. *halophilus*, suggests their presence in solar salt and their proliferation during the fermentation of *doenjang*. However, we are unaware of studies designed to identify the predominant species of *doenjang* derived from solar salt. Here we investigated bacterial migration using culture-independent and -dependent analyses. These results delineate the characteristics of bacterial migration into *doenjang* from its components and suggest the involvement of diverse bacterial species.

## Materials and methods

### *Meju*, solar salt, and *doenjang*

*Meju*, solar salt, and *doenjang* samples were purchased from a traditional manufacturer in the Seosan area of Korea on different timetables [11]. *Meju*, fermented soybean for 2 months, and solar salt were used materials of purchased *doenjang* which was fermented for 3 months. Samples were ground, homogenized, or both, with an equal amount of sterilized water, then filtered through sterilized cheesecloth. The filtrates were analyzed for NaCl concentration, pH values, bacterial compositions, and for their culture-independent bacterial communities. NaCl concentration was measured using silver nitrate according to the Mohr method [12], and pH was measured using a pH meter. Microbial counts were determined by spreading appropriate dilutions of the filtrates on Plate Count Agar (PCA) (BD Difco, Detroit, MI, USA), and PCA containing 7% or 12% NaCl as a final concentration.

### Pyrosequencing of culture-independent samples

To perform a culture-independent metagenomics analysis of the bacterial community, a DNeasy PowerSoil Kit (Qiagen, Hilden, Germany) was used to prepare DNA from filtrates of *meju*, solar salt, and *doenjang*. DNA quantity and quality were determined using PicoGreen and a Nanodrop. The V3 to V4 hypervariable regions of the bacterial 16S rRNA gene from genomic DNA were amplified using the primers as follows: forward, 5′- TCG TCG GCA GCG TCA GAT GTG TAT AAG AGA CAG CCT ACG GGN GGC WGC AG -3′ and reverse, 5′- GTC TCG TGG GCT CGG AGA TGT GTA TAA GAG ACA GGA CTA CHV GGG TAT CTA ATC C -3′) using a MyCycler Thermal Cycler (Bio-Rad, USA). PCR mixtures contained 30 ng of genomic DNA, 50 pM each primer, and Han-Taq polymerase (Genenmed, Korea). PCR was performed as follows: initial denaturation at 94 °C for 90 s, 30 cycles of denaturation at 94 °C for 45 s, annealing at 55 °C for 45 s, extension at 72 °C for 45 s, and final extension at 72 °C for 5 min.

Each primer was concatenated to an 8-base sample-specific barcode sequence and a common linker sequence (TC, forward primer and CA, reverse primer) at the 5′ terminus [13]. Pyrosequencing was performed by Macrogen (Korea) using the MiSeq platform (Illumina, San Diego, USA) according to the manufacturer’s instructions. To assess richness estimators of bacterial species, diversity indices, and rarefaction curves, we applied the pyrosequencing pipeline of the Ribosomal RNA Database Project [14]. Venn diagrams were generated using the packages gplots and limma in R v.4.0.0 (http://www.R-projet.org).

### Identification of culture-dependent isolates using 16S rRNA gene sequence analysis

Culture media used to isolate bacteria were as follows: MRS agar (BD Difco), MRS agar supplemented with 7% or 10% NaCl, Nutrient Agar (BD Difco), and Nutrient Agar containing 7% or 12% NaCl. All media were incubated at 30 °C until distinguishable colonies appeared, and >20 types of colonies were collected from each plate according to differences in morphology, growth characteristics, and the number of colonies. The colonies were purified through successive transfers to plates containing the same type of agar medium used for isolation.

Genomic DNAs of isolates were extracted using a DNeasy Blood & Tissue Kit (Qiagen, Germany). Amplification of the 16S rRNA gene was performed using eubacterial universal primers 27F (5′-AGA GTT TGA TCC TGG CTC AG-3′) and 1492R (5′-GGT TAC CTT GTT ACG ACT T-3′) [15] with a T3000 Thermocycler (Biometra, Germany). The PCR mixture comprised template DNA (10 ng), 0.5 mM each primer, 1 U of *Taq* polymerase (ImClone, Daejeon, Korea), 10 mM dNTPs, and 2.5 mM MgCl_2_. Samples were heated for 5 min at 95 °C and then amplified as follows: 30 cycles for 1 min at 95 °C, 1 min at 58 °C, and 1 min at 72 °C. The PCR products were purified and sequenced using a custom service provided by Bionics Co. (Seoul, Korea). We used a web-hosted BLASTn algorithm to query the National Center for Biotechnology Information database for 16S rRNA gene sequences (http://blast.ncbi.nlm.nih.gov). The phylogenetic relationships of the isolates were inferred according to this analysis.

## Results and discussion

### Physicochemical analysis and viable counts

*Meju* and *doenjang* samples had average NaCl concentrations of 2.1%, and 13.9%, respectively (Table 1). The salt contents of *meju* and *doenjang* were similar to those acquired from other regions [5], but differed in pH values. The salt concentrations and pH values of solar salt preparations vary [16], and in the present study, the salt content was 69.2% (pH 4.87).

**Table 1:** pH values and NaCl concentrations of *meju* and *doenjang*.

Sample	*Meju*	Solar salt	*Doenjang*
pH	6.93 ± 0.12	4.87 ± 0.09	6.18 ± 0.03
NaCl concentration (%)	2.1 ± 0.1	69.2 ± 0.4	13.9 ± 0.5

The results represent the average values of three replicates.

The average concentrations of the bacterial populations of *meju* and *doenjang*, determined using PCA, were 7.8 × 10^8^ CFU/g and 6.7 × 10^8^ CFU/g, respectively, and 1.1 × 10^3^ CFU/g and 7.5 × 10^3^ CFU/g using PCA supplemented with 12% NaCl (Table 2). The NaCl concentration of doenjang was approximately 12%, thus these results suggest that the high NaCl concentration of *doenjang* inhibited bacterial growth during fermentation. In solar salt, there were few viable bacteria (averages of 4.3 × 10^1^ CFU/g and 2.5×10^0^ CFU/g on PCA and PCA with 12% NaCl, respectively). The cell counts were greatly influenced by the NaCl concentrations of the plating media.

**Table 2:** Effect of NaCl concentrations on viable bacterial counts.

NaCl concentration	Cell count (CFU/g)		
	*Meju*	Solar salt	*Doenjang*
0%	7.8 × 10^8^ ± 1.1 × 10^7^	4.3 × 10^1^ ± 1.1 × 10^1^	6.7 × 10^8^ ± 7.4 × 10^7^
7%	4.6 × 10^8^ ± 5.7 × 10^7^	2.5 × 10^1^ ± 7.1	7.7 × 10^8^ ± 4.9 × 10^7^
12%	1.1 × 10^3^ ± 1.3 × 10^3^	2.5 ± 0.0	7.5 × 10^3^ ± 6.0 × 10^2^

Bacteria were plated on plate-count agar (PCA).

### Bacterial communities identified using culture-independent analysis

Analyses of *meju*, solar salt, and *doenjang* yielded 190,634; 202,956; and 123,972 sequences of sufficient quality, respectively. The sequence coverage of these samples ranged from 0.99 to 1.00, which provided sufficient statistical power to conduct analyses of bacterial communities. The numbers of operational taxonomic units and Chao1 (species richness), and Shannon indexes (species diversity) are shown in Table 3. The Chao1 and Shannon indexes ranged from 30–351 and 0.65–5.68, respectively, indicating that the species in solar salt were more diverse than those of *doenjang* and *meju*.

**Table 3:** Microbial diversity indices of *meju*, solar salt, and *doenjang*.

Sample name	High-quality reads	OTUs	Chao1	Shannon	Goods Coverage
*Meju*	190,634	63	101.5	0.65	0.99
Solar salt	202,956	351	351	5.68	1.00
*Doenjang*	123,972	30	30	2.03	1.00

**Fig 1:**
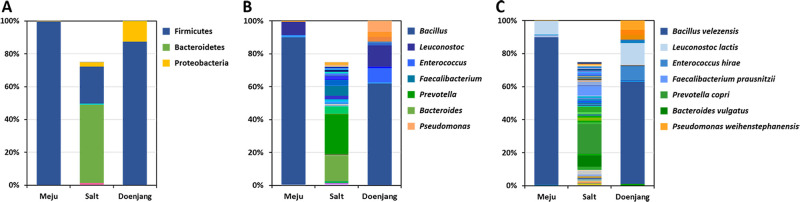
Bacterial species compositions of *meju* and *doenjang*. Data portray phylum- (A), genus- (B), and species- (C) levels of the V3/V4 regions of 16S rRNA gene sequences. Regions >200 bp were classified using the CD-HIT-OUT (97% confidence threshold). Categories of relative abundance >5% are shown.

The phylogenetic classification of bacterial communities is summarized in Fig 1 and S1 Table. Members of *Firmicutes* (99.57%), *Proteobacteria* (0.28%), *Bacteroidetes* (0.13%), and *Actinobacteria* (0.02%) were identified in *meju*, and members of *Firmicutes* (87.36%), *Proteobacteria* (12.58%), and *Actinobacteria* (0.06%) were identified in *doenjang*. Among those identified in solar salt, the most abundant were *Bacteroidetes* (47.75%), *Firmicutes* (22.59%), *Proteobacteria* (2.45%), and *Actinobacteria* (1.32%), which represented 74.88% of the bacterial composition. *Euryarchaeota* (archaea) represented 23.90% of the population.

The predominant genera in *meju* were *Bacillus* (89.57%), *Leuconostoc* (7.88%), and *Clostridium* (1.18%). The predominant genera in *doenjang* were *Bacillus* (61.75%), *Leuconostoc* (12.96%), *Enterococcus* (8.85%), and *Pseudomonas* (6.40%). In solar salt, the predominant genera were *Prevotella* (24.00%) and *Bacteroides* (15.31%).

The predominant species in *meju* were *Bacillus velezensis* (89.55%), *Leuconostoc lactis* (7.88%), and *Clostridium saccharoperbutylactonicum* (1.17%). The predominant species in *doenjang* were *B*. *velezensis* (61.74%), *L*. *lactis* (12.95%), and *Enterococcus hirae* (8.84%). In solar salt, the predominant species were *Prevotella copri* (17.98%) and *Bacteroides vulgatus* (6.39%). Culture-independent analysis of Nam et al. [8] exhibited that microbial community of traditional *doenjang* were diverse in different regions. Current microbial communities did not perfectly matched with those of traditional samples [6–10]. Therefore, current results exhibited the microbial communities of *doenjang* including *meju* and solar salt in Seosan region of Korea, not whole traditional fermented soybean.

**Fig 2:**
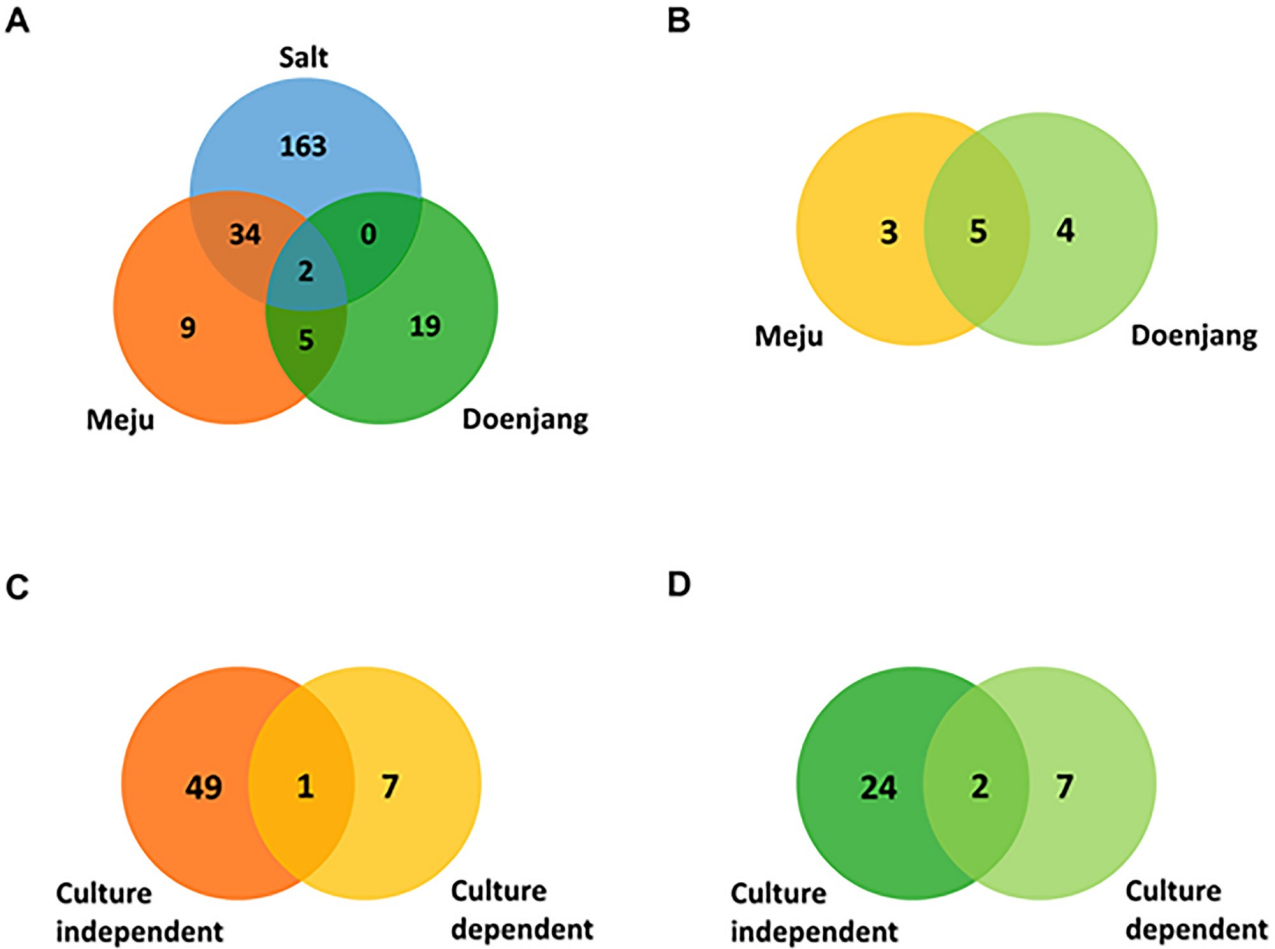
Proportional Venn diagram showing the distribution of species. Venn diagrams of the results of culture-independent analysis (A), culture-dependent analysis (B), *meju* (C), and *doenjang* (D), generated using the gplots and limma packages in R. Overlapping regions represent species shared between analytical methods, samples, or both. The numbers outside the overlapping regions indicate the numbers of species.

The species pools shared by the bacterial communities in *meju*, solar salt, and *doenjang* are depicted in Fig 2A. Three samples include *B*. *velezensis* and *Erwinia aphidicola*, which accounted for 89.59%, 0.05%, 64.51% of the species in *meju*, solar salt, and *doenjang*, respectively. Except for two species, the others were not shared between solar salt and *doenjang*. These results indicate that solar salt had an insignificant effect on the composition of the bacterial communities in *doenjang*. Notably the seven species shared between *meju* and *doenjang* accounted for 98.88% and 87.62% of their populations, respectively. These results indicate that most bacterial communities of *meju* populated *doenjang*.

### Bacterial communities identified using culture-dependent analysis

The most frequently identified bacterial species in solar salt were not consistent with those of *meju* and *doenjang*. In addition, viable bacteria from solar salt did not obtained on nutrient and MRS by culture-dependent analysis. Therefore, we conducted culture-dependent approaches, which identified 130 and 123 species in *meju* and *doenjang* samples, respectively (Table 4). Bacilli formed colonies on nutrient and MRS agar independent of NaCl concentration, and lactic acid bacteria (LAB) were isolated using MRS agar. The addition of NaCl to MRS decreased the numbers of LAB, *E*. *faecium*, and *Leuconostoc mesenteroides*, while halotolerant LAB and *T*. *halophilius* formed colonies on MRS containing 10% NaCl. Furthermore, eight species of *Bacillus*; the LABs *E*. *faecium*, *L*. *mesenteroides*, and *T*. *halophilus*; and one staphylococcal were identified. As the NaCl concentration increased, changed from *E*. *faecium* to *T*. *halophilus*, while the number of bacillus species did not change. *Bacillus* species were the most abundant in *meju* and *doenjang*, and the population of *B*. *velezensis* was the largest.

**Table 4:** Numbers of bacterial species isolated from *meju* and *doenjang*.

Species	*Meju*						*Doenjang*						Total
	MRS			Nutrient			MRS			Nutrient			
	0%^a^	7%	10%	0%	7%	12%	0%	7%	10%	0%	7%	12%	
*Bacillus amyloliquefaciens*		1			2			1	1	1	2		8
*Bacillus atrophaeus*												1	1
*Bacillus coagulans*							1						1
*Bacillus licheniformis*												1	1
*Bacillus pumilus*												1	1
*Bacillus siamensis*		1			2			2		1			6
*Bacillus subtilis*			2		1	1		3	1	1	1		10
*Bacillus velezensis*		23	3	17	20	29	20	22	1	17	20	19	191
*Enterococcus faecium*	19			4									23
*Leuconostoc mesenteroides*	2												2
*Tetragenococcus halophilus*			2						6				8
*Staphylococcus saprophyticus*			1										1
**Total**	21	25	8	21	25	30	21	28	9	20	23	22	253

^a^Percentages indicate the final concentrations of NaCl in the media.

*E*. *faecium*, which was the predominant species in *meju*, was not isolated from *doenjang* and did not grow in medium containing 7% NaCl. These data agree with a previous report that the optimum concentration of NaCl for growth of *Enterococcus* is 6.5% [17]. Therefore, these results support the conclusion that the high salt concentration of *doenjang* inhibited the growth of *E*. *faecium*.

*T*. *halophilus* was isolated from *doenjang* and *meju* using media containing NaCl. Other investigators suggest that the halophile *T*. *halophilus* migrates into *doenjang* from solar salt during the brining step [9,10]. Conversely, our culture-independent method did not detect *T*. *halophilus* in solar salt (S1 Table), and *T*. *halophilus* was isolated from *meju* and *doenjang* using culture-dependent methods (Table 4). Therefore, we carefully suggest that *T*. *halophilus* migrated from *meju* to *doenjang*.

*S*. *saprophyticus* was isolated here only from *meju* using media containing NaCl, although *meju* samples contain 2.1% NaCl. Our previous studies found that coagulase-negative staphylococci cultures containing *S*. *saprophyticus* are predominantly detected in *meju* and *doenjang* and contribute to its enhanced sensory properties [18–20]. However, only *S*. *saprophyticus* was detected in the present study.

Here we show that the species shared by the bacterial communities of *meju*, and *doenjang* were as follows: *B*. *amyloliquefaciens*, *B*. *siamensis*, *B*. *subtilis*, *B*. *velezensis*, and *T*. *halophilus* (Fig 2B). These five shared species accounted for 80.00%, and 96.75% of those in *meju* and *doenjang*, respectively. However, previous our culture-dependent studies showed that shared species and ratio were diverse [5]. High shared rate of species in current study should be derived from salt-tolerance *B*. *velezensis*, which species were detected dominantly in *meju*, while salt-sensitive species such as *Enterococcus faecium* were detected dominantly from *meju* in previous study and thus those species might be not survived in high-salt condition, *doenjang* [5]. Consequently, culture-dependent results revealed that the bacterial communities of *doenjang* were derived from *meju* and that the high-salt concentration of *doenjang* accounted for the differences in their bacterial populations.

### Comparison of bacterial communities identified using culture-dependent and culture-independent analyses

**Fig 3:**
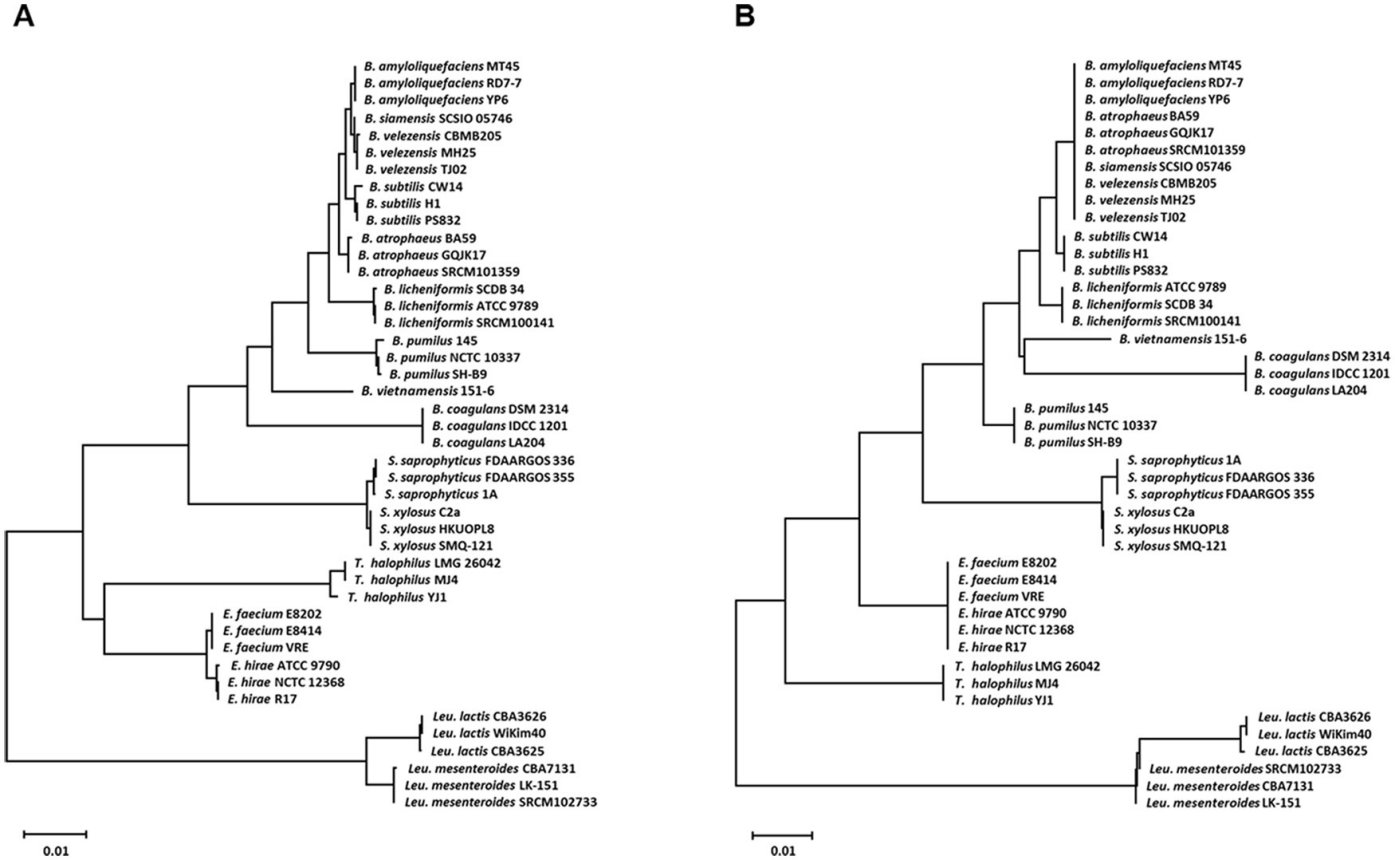
Phylogenetic tree of bacterial 16S rRNA gene sequences identified in *meju* and *doenjang*. Maximum-likelihood phylogenetic trees show the relationships of the sequences of the entire 16S rRNA gene (A) and its V3 to V4 hypervariable regions (B). Branches with bootstrap values <50% are collapsed.

Culture-independent analysis showed greater diversity in bacterial communities compared with culture-dependent analysis (Fig 2C and 2D). Between two methods, although the number of shared bacterial species was low, the portions of shared species were high. For example, in *meju*, 50 and 8 species were detected using culture-independent and -dependent analyses, respectively (Fig 2C), and only one species (*B*. *velezensis*) was identified using both techniques, which accounted for 89.55% and 66.15% of the analyses, respectively. Eight species were uniquely detected using the culture-dependent technique, and four of eight species were *B*. *amyloliquefaciens*, *B*. *siamensis*, *B*. *subtilis*, and *B*. *velezensis*, which share >99% identities among their 16S rRNA gene sequences. Interestingly, the sequences of the V3 to V4 hypervariable regions of did not distinguish *B*. *amyloliquefaciens*, *B*. *siamensis*, and *B*. *velezensis* from each other, compared with those of the entire 16S rRNA gene (Fig 3). Similarly, *E*. *faecium* and *E*. *hirae* were not distinguished according to the sequences of their V3 to V4 hypervariable regions. We assumed that although *B*. *velezensis* was annotated according to the pyrosequencing data, the population may change from *B*. *velezensis* to *B*. *amyloliquefaciens* or *B*. *siamensis* as well as *E*. *hirae*, which annotated according to the pyrosequencing analysis, may be replaced by *E*. *faecium*.

Here we identified 26 and 9 species in *doenjang* using culture-independent and culture-dependent analyses, respectively (Fig 2D); and *B*. *velezensis* and *T*. *halophilus* were identified by each. These species accounted for 62.66% and 85.27% of those identified by culture-independent and -dependent analysis, respectively. Eight bacilli were detected using the culture-dependent assay, while only one was detected using the culture-independent assay. These findings indicate that culture-dependent analysis was more precise and that the findings of culture-independent analysis and pyrosequencing included those of culture-dependent analysis.

The present study reveals the advantages and limitations of the two types of analyses. The former serves as a powerful tool to evaluate microbial diversity. Although the latter method identifies viable cells, which are used to screen for starter candidates, it does not sufficiently assess bacterial diversity. Therefore, the complementary results using the two methods here may likely be explained by bacterial migration from *meju* into *doenjang*. Our present results show that most predominant bacterial species of *meju* may migrate into *doenjang* during fermentation.

Consistent with previous studies, here we identified *Bacillus* as the major bacterial genus [5,7,21–24]. Among them, *B*. *velezensis* was the predominant species in *meju* and *doenjang* (Table 3 and Fig 1). Phylogenetic analysis reclassified *B*. *velezensis* as a synonym of *B*. *methylotrophicus*, *B*. *amyloliquefaciens* subsp. *plantarum*, and *B*. *oryzicola* [25]. These species are typically detected in *meju* and *doenjang* using culture-dependent and culture-independent analyses. *B*. *velezensis* is widely detected in sources such as soil, wheat anthers, and rivers [26–28]; and it is used as a biocontrol agent in agriculture [28–30]. Furthermore, these species are frequently detected in fermented foods containing *meju* and *doenjang* [24,31,32]. We conclude therefore that *B*. *velezensis* may be transferred from environments such as soil to *meju*, where it becomes a dominant species in during fermentation and then migrates into *doenjang* [24,31,32].

In the present study, the culture-independent method most frequently identified *L*. *lactis* in *meju* and *doenjang*. However, *L*. *lactis* was not isolated using culture-dependent methods. *Leuconostoc* is the dominant genus of kimchi, the traditional Korean fermented kimchi cabbage, which is used as a commercial starter for kimchi fermentation [33]. However, this genus is rarely detected as a minor group in high-salt fermented foods such as *doenjang* and *jeotgal* using culture-dependent approaches. Culture-independent analyses such as PCR-denaturing gradient gel electrophoresis found that the LAB population, including *Leuconostoc*, is dominant in *meju* and *doenjang* [7,34,35]. However, we show here that most LAB were not sufficiently salt-tolerant to proliferate in *doenjang*. Furthermore, we did not detect *E*. *faecium* and *L*. *mesenteroides* using media containing NaCl (Table 4), consistent with the results of a previous study [5]. Therefore, culture and pyrosequencing results indicate that LAB may be present in *meju*.

We believe that the present study is the most comprehensive comparison of culture-independent and -dependent methods used to track bacterial migration from *meju* into *doenjang*. Although the bacterial populations identified using culture-dependent analysis were less diverse than with the culture-independent analysis, the latter identified the predominant bacterial species. This suggests that novel culturing techniques are required to identify process-relevant bacteria and to develop starter candidates. Although the use of pyrosequencing wildly outpaces culture-dependent analysis, more accurate species identification must be achieved. Moreover, the complementary nature of two methods should increase our understanding of bacterial migration and identify novel bacteria that are present in high-salt fermented foods.

## Supporting information

S1 Table Taxonomic classification at the species levels showing the bacterial communities of *meju*, solar salt, and *doenjang*. (DOCX)
