# Supplementary material for: Culture-dependent and -independent investigations of bacterial migration into doenjang from its components meju and solar salt
Source: PLoS One. 2020 Oct 13;15(10):e0239971. doi: 10.1371/journal.pone.0239971 (PMC7553307; doi:10.1371/journal.pone.0239971)
Supplement: S1 Table — (DOCX) [file pone.0239971.s001.docx]

**SUPPLEMENTAL MATERIAL**

**Culture-Dependent and -Independent Investigations of Bacterial Migration into *Doenjang* from its Components *Meju* and Solar Salt**

Jung-Min Lee^1†^, Sojeong Heo^1†^, Yoon-Su Kim^1^, Jong-Hoon Lee^2^, Do-Won Jeong^1*^

^†^These authors contributed equally to this work.

**S1 Table. Taxonomic classification at the species levels showing bacterial community of *meju*, solar salt, and *doenjang*.**

| **Phylum** | **Genus** | **Species** | ***Meju*** | **Solar salt** | ***Doenjang*** |
| --- | --- | --- | --- | --- | --- |
| Actinobacteria | *Arthrobacter* | *Arthrobacter halodurans* | 0.028 | 0.000 | 0.024 |
|  | *Bifidobacterium* | *Bifidobacterium adolescentis* | 0.000 | 0.559 | 0.000 |
|  | *Bifidobacterium* | *Bifidobacterium longum* | 0.000 | 0.292 | 0.000 |
|  | *Bifidobacterium* | *Bifidobacterium pseudolongum* | 0.000 | 0.030 | 0.000 |
|  | *Collinsella* | *Collinsella aerofaciens* | 0.000 | 0.410 | 0.000 |
|  | *Curtobacterium* | *Curtobacterium oceanosedimentum* | 0.000 | 0.000 | 0.027 |
|  | *Demequina* | *Demequina lutea* | 0.000 | 0.006 | 0.000 |
|  | *Gaiella* | *Gaiella occulta* | 0.000 | 0.006 | 0.000 |
|  | *Kocuria* | *Kocuria dechangensis* | 0.000 | 0.000 | 0.010 |
|  | *Stenotrophobacter* | *Stenotrophobacter roseus* | 0.000 | 0.006 | 0.000 |
|  | *Streptomyces* | *Streptomyces lavendulae* | 0.000 | 0.006 | 0.000 |
| Bacteroidetes | *Alistipes* | *Alistipes indistinctus* | 0.000 | 0.028 | 0.000 |
|  | *Alistipes* | *Alistipes onderdonkii* | 0.000 | 0.028 | 0.000 |
|  | *Alistipes* | *Alistipes putredinis* | 0.003 | 0.963 | 0.000 |
|  | *Alistipes* | *Alistipes shahii* | 0.000 | 0.248 | 0.000 |
|  | *Bacteroides* | *Bacteroides caccae* | 0.006 | 0.527 | 0.000 |
|  | *Bacteroides* | *Bacteroides caecigallinarum* | 0.001 | 0.683 | 0.000 |
|  | *Bacteroides* | *Bacteroides cellulosilyticus* | 0.001 | 0.143 | 0.000 |
|  | *Bacteroides* | *Bacteroides coprocola* | 0.000 | 0.463 | 0.000 |
|  | *Bacteroides* | *Bacteroides coprophilus* | 0.000 | 0.021 | 0.000 |
|  | *Bacteroides* | *Bacteroides fragilis* | 0.000 | 0.546 | 0.000 |
|  | *Bacteroides* | *Bacteroides massiliensis* | 0.004 | 0.591 | 0.000 |
|  | *Bacteroides* | *Bacteroides mediterraneensis* | 0.000 | 0.019 | 0.000 |
|  | *Bacteroides* | *Bacteroides nordii* | 0.000 | 0.006 | 0.000 |
|  | *Bacteroides* | *Bacteroides ovatus* | 0.003 | 1.163 | 0.000 |
|  | *Bacteroides* | *Bacteroides paurosaccharolyticus* | 0.000 | 0.006 | 0.000 |
|  | *Bacteroides* | *Bacteroides plebeius* | 0.022 | 2.452 | 0.000 |
|  | *Bacteroides* | *Bacteroides stercoris* | 0.000 | 0.160 | 0.000 |
|  | *Bacteroides* | *Bacteroides thetaiotaomicron* | 0.000 | 0.245 | 0.000 |
|  | *Bacteroides* | *Bacteroides uniformis* | 0.004 | 1.885 | 0.000 |
|  | *Bacteroides* | *Bacteroides vulgatus* | 0.007 | 6.392 | 0.000 |
|  | *Barnesiella* | *Barnesiella intestinihominis* | 0.001 | 0.175 | 0.000 |
|  | *Butyricimonas* | *Butyricimonas virosa* | 0.001 | 0.021 | 0.000 |
|  | *Coprobacter* | *Coprobacter fastidiosus* | 0.000 | 0.013 | 0.000 |
|  | *Fermentimonas* | *Fermentimonas caenicola* | 0.000 | 0.041 | 0.000 |
|  | *Lentimicrobium* | *Lentimicrobium saccharophilum* | 0.000 | 0.006 | 0.000 |
|  | *Mariniphaga* | *Mariniphaga anaerophila* | 0.000 | 0.006 | 0.000 |
|  | *Massiliprevotella* | *Massiliprevotella massiliensis* | 0.000 | 0.006 | 0.000 |
|  | *Natronoflexus* | *Natronoflexus pectinivorans* | 0.000 | 0.009 | 0.000 |
|  | *Odoribacter* | *Odoribacter splanchnicus* | 0.000 | 0.053 | 0.000 |
|  | *Parabacteroides* | *Parabacteroides distasonis* | 0.000 | 0.361 | 0.000 |
|  | *Parabacteroides* | *Parabacteroides goldsteinii* | 0.000 | 0.036 | 0.000 |
|  | *Parabacteroides* | *Parabacteroides johnsonii* | 0.000 | 0.009 | 0.000 |
|  | *Parabacteroides* | *Parabacteroides merdae* | 0.000 | 0.521 | 0.000 |
|  | *Paraprevotella* | *Paraprevotella clara* | 0.000 | 0.034 | 0.000 |
|  | *Portibacter* | *Portibacter lacus* | 0.000 | 0.019 | 0.000 |
|  | *Prevotella* | *Prevotella bivia* | 0.000 | 0.043 | 0.000 |
|  | *Prevotella* | *Prevotella brevis* | 0.000 | 0.006 | 0.000 |
|  | *Prevotella* | *Prevotella copri* | 0.045 | 17.975 | 0.000 |
|  | *Prevotella* | *Prevotella disiens* | 0.003 | 1.112 | 0.000 |
|  | *Prevotella* | *Prevotella histicola* | 0.010 | 1.012 | 0.000 |
|  | *Prevotella* | *Prevotella loescheii* | 0.012 | 2.028 | 0.000 |
|  | *Prevotella* | *Prevotella oralis* | 0.000 | 0.026 | 0.000 |
|  | *Prevotella* | *Prevotella stercorea* | 0.001 | 0.978 | 0.000 |
|  | *Prevotella* | *Prevotella timonensis* | 0.000 | 0.822 | 0.000 |
|  | *Prevotellamassilia* | *Prevotellamassilia timonensis* | 0.000 | 0.233 | 0.000 |
|  | *Salinibacter* | *Salinibacter altiplanensis* | 0.000 | 1.095 | 0.000 |
|  | *Salinibacter* | *Salinibacter ruber* | 0.000 | 0.011 | 0.000 |
|  | *Salinibacter* | *Salinibacter iranicus* | 0.000 | 3.287 | 0.000 |
|  | *Sphingobacterium* | *Sphingobacterium thermophilum* | 0.000 | 0.043 | 0.000 |
|  | *Sunxiuqinia* | *Sunxiuqinia rutila* | 0.000 | 0.006 | 0.000 |
|  | *Tangfeifania* | *Tangfeifania diversioriginum* | 0.000 | 0.006 | 0.000 |
| Chloroflexi | *Pelolinea* | *Pelolinea submarina* | 0.000 | 0.030 | 0.000 |
| Cyanobacteria | *Aerosakkonema* | *Aerosakkonema funiforme* | 0.001 | 0.542 | 0.000 |
| Firmicutes | *Acetanaerobacterium* | *Acetanaerobacterium elongatum* | 0.000 | 0.028 | 0.000 |
|  | *Acidaminococcus* | *Acidaminococcus fermentans* | 0.000 | 0.348 | 0.000 |
|  | *Agathobaculum* | *Agathobaculum desmolans* | 0.000 | 0.009 | 0.000 |
|  | *Alkalibaculum* | *Alkalibaculum bacchi* | 0.000 | 0.013 | 0.000 |
|  | *Allisonella* | *Allisonella histaminiformans* | 0.000 | 0.013 | 0.000 |
|  | *Anaerococcus* | *Anaerococcus mediterraneensis* | 0.000 | 0.292 | 0.000 |
|  | *Anaerococcus* | *Anaerococcus octavius* | 0.000 | 0.006 | 0.000 |
|  | *Anaerococcus* | *Anaerococcus vaginalis* | 0.001 | 0.006 | 0.000 |
|  | *Anaeromassilibacillus* | *Anaeromassilibacillus senegalensis* | 0.000 | 0.006 | 0.000 |
|  | *Anaerosporobacter* | *Anaerosporobacter mobilis* | 0.243 | 0.028 | 0.000 |
|  | *Anaerostipes* | *Anaerostipes hadrus* | 0.000 | 0.036 | 0.000 |
|  | *Anaerotignum* | *Anaerotignum aminivorans* | 0.000 | 0.021 | 0.000 |
|  | *Anaerovibrio* | *Anaerovibrio lipolyticus* | 0.000 | 0.075 | 0.000 |
|  | *Bacillus* | *Bacillus aerius* | 0.009 | 0.000 | 0.000 |
|  | *Bacillus* | *Bacillus nanhaiisediminis* | 0.000 | 0.000 | 0.010 |
|  | *Bacillus* | *Bacillus velezensis* | 89.549 | 0.030 | 61.740 |
|  | *Bacillus* | *Bacillus vietnamensis* | 0.009 | 0.000 | 0.000 |
|  | *Blautia* | *Blautia faecis* | 0.000 | 0.111 | 0.000 |
|  | *Blautia* | *Blautia wexlerae* | 0.000 | 0.032 | 0.000 |
|  | *Butyricicoccus* | *Butyricicoccus pullicaecorum* | 0.000 | 0.009 | 0.000 |
|  | *Caloramator* | *Caloramator australicus* | 0.000 | 0.021 | 0.000 |
|  | *Caloramator* | *Caloramator fervidus* | 0.000 | 0.009 | 0.000 |
|  | *Catenibacterium* | *Catenibacterium mitsuokai* | 0.000 | 0.036 | 0.000 |
|  | *Christensenella* | *Christensenella massiliensis* | 0.000 | 0.120 | 0.000 |
|  | *Clostridioides* | *Clostridioides mangenotii* | 0.000 | 0.000 | 0.010 |
|  | *Clostridium* | *Clostridium asparagiforme* | 0.000 | 0.006 | 0.000 |
|  | *Clostridium* | *Clostridium cellulolyticum* | 0.000 | 0.006 | 0.000 |
|  | *Clostridium* | *Clostridium clostridioforme* | 0.000 | 0.141 | 0.000 |
|  | *Clostridium* | *Clostridium colinum* | 0.000 | 0.006 | 0.000 |
|  | *Clostridium* | *Clostridium combesii* | 0.000 | 0.000 | 0.643 |
|  | *Clostridium* | *Clostridium hathewayi* | 0.000 | 0.009 | 0.000 |
|  | *Clostridium* | *Clostridium hungatei* | 0.000 | 0.013 | 0.000 |
|  | *Clostridium* | *Clostridium intestinale* | 0.000 | 0.000 | 0.010 |
|  | *Clostridium* | *Clostridium lactatifermentans* | 0.000 | 0.023 | 0.000 |
|  | *Clostridium* | *Clostridium leptum* | 0.000 | 0.006 | 0.000 |
|  | *Clostridium* | *Clostridium methylpentosum* | 0.000 | 0.137 | 0.000 |
|  | *Clostridium* | *Clostridium papyrosolvens* | 0.000 | 0.060 | 0.000 |
|  | *Clostridium* | *Clostridium saccharogumia* | 0.000 | 0.006 | 0.000 |
|  | *Clostridium* | *Clostridium saccharoperbutylacetonicum* | 1.175 | 0.000 | 0.044 |
|  | *Clostridium* | *Clostridium saudiense* | 0.000 | 0.538 | 0.000 |
|  | *Clostridium* | *Clostridium spiroforme* | 0.000 | 0.006 | 0.000 |
|  | *Clostridium* | *Clostridium straminisolvens* | 0.000 | 0.032 | 0.000 |
|  | *Clostridium* | *Clostridium viride* | 0.000 | 0.006 | 0.000 |
|  | *Clostridium* | *Clostridium xylanolyticum* | 0.001 | 0.220 | 0.000 |
|  | *Coprococcus* | *Coprococcus catus* | 0.000 | 0.006 | 0.000 |
|  | *Coprococcus* | *Coprococcus eutactus* | 0.000 | 0.425 | 0.000 |
|  | *Dethiobacter* | *Dethiobacter alkaliphilus* | 0.000 | 0.021 | 0.000 |
|  | *Dialister* | *Dialister invisus* | 0.000 | 0.256 | 0.000 |
|  | *Dialister* | *Dialister succinatiphilus* | 0.001 | 0.681 | 0.000 |
|  | *Dorea* | *Dorea formicigenerans* | 0.000 | 0.006 | 0.000 |
|  | *Dorea* | *Dorea longicatena* | 0.000 | 0.006 | 0.000 |
|  | *Enterococcus* | *Enterococcus hirae* | 0.205 | 0.000 | 8.838 |
|  | *Enterococcus* | *Enterococcus lactis* | 0.000 | 0.000 | 0.010 |
|  | *Ethanoligenens* | *Ethanoligenens harbinense* | 0.000 | 0.006 | 0.000 |
|  | *Clostridium* | *Eubacterium combesii* | 0.274 | 0.000 | 0.000 |
|  | *Eubacterium* | *Eubacterium coprostanoligenes* | 0.000 | 0.807 | 0.000 |
|  | *Eubacterium* | *Eubacterium eligens* | 0.001 | 0.034 | 0.000 |
|  | *Eubacterium* | *Eubacterium rectale* | 0.000 | 1.133 | 0.000 |
|  | *Eubacterium* | *Eubacterium ruminantium* | 0.000 | 0.038 | 0.000 |
|  | *Eubacterium* | *Eubacterium siraeum* | 0.001 | 0.177 | 0.000 |
|  | *Faecalibacterium* | *Faecalibacterium prausnitzii* | 0.018 | 5.942 | 0.000 |
|  | *Faecalimonas* | *Faecalimonas umbilicata* | 0.000 | 0.006 | 0.000 |
|  | *Fastidiosipila* | *Fastidiosipila sanguinis* | 0.000 | 0.006 | 0.000 |
|  | *Flavonifractor* | *Flavonifractor plautii* | 0.000 | 0.006 | 0.000 |
|  | *Flintibacter* | *Flintibacter butyricus* | 0.000 | 0.265 | 0.000 |
|  | *Frisingicoccus* | *Frisingicoccus caecimuris* | 0.000 | 0.006 | 0.000 |
|  | *Fusicatenibacter* | *Fusicatenibacter saccharivorans* | 0.000 | 0.128 | 0.000 |
|  | *Gelria* | *Gelria glutamica* | 0.000 | 0.015 | 0.000 |
|  | *Gemmiger* | *Gemmiger formicilis* | 0.001 | 2.224 | 0.000 |
|  | *Gracilibacter* | *Gracilibacter thermotolerans* | 0.000 | 0.508 | 0.000 |
|  | *Herbivorax* | *Herbivorax saccincola* | 0.000 | 0.032 | 0.000 |
|  | *Intestinibacter* | *Intestinibacter bartlettii* | 0.000 | 0.006 | 0.000 |
|  | *Intestinimonas* | *Intestinimonas butyriciproducens* | 0.000 | 0.303 | 0.000 |
|  | *Kineothrix* | *Kineothrix alysoides* | 0.000 | 0.079 | 0.000 |
|  | *Lachnoclostridium* | *Lachnoclostridium pacaense* | 0.000 | 0.228 | 0.000 |
|  | *Lachnospira* | *Lachnospira pectinoschiza* | 0.000 | 0.006 | 0.000 |
|  | *Lacrimispora* | *Lacrimispora xylanolytica* | 0.000 | 0.000 | 0.534 |
|  | *Lactobacillus* | *Lactobacillus animalis* | 0.000 | 0.662 | 0.000 |
|  | *Lactobacillus* | *Lactobacillus johnsonii* | 0.000 | 0.613 | 0.000 |
|  | *Lactobacillus* | *Lactobacillus reuteri* | 0.003 | 0.173 | 0.000 |
|  | *Lactobacillus* | *Lactobacillus rogosae* | 0.001 | 0.440 | 0.000 |
|  | *Lactobacillus* | *Lactobacillus ruminis* | 0.000 | 0.006 | 0.000 |
|  | *Lactobacillus* | *Lactobacillus sakei* | 0.000 | 0.000 | 0.333 |
|  | *Lactobacillus* | *Lactobacillus ultunensis* | 0.000 | 0.126 | 0.000 |
|  | *Leuconostoc* | *Leuconostoc lactis* | 7.877 | 0.000 | 12.952 |
|  | *Leuconostoc* | *Leuconostoc mesenteroides* | 0.000 | 0.000 | 0.010 |
|  | *Megamonas* | *Megamonas funiformis* | 0.000 | 0.231 | 0.000 |
|  | *Megasphaera* | *Megasphaera elsdenii* | 0.000 | 0.196 | 0.000 |
|  | *Mitsuokella* | *Mitsuokella jalaludinii* | 0.000 | 0.006 | 0.000 |
|  | *Mitsuokella* | *Mitsuokella multacida* | 0.000 | 0.013 | 0.000 |
|  | *Muribaculum* | *Muribaculum intestinale* | 0.001 | 1.193 | 0.000 |
|  | *Natranaerovirga* | *Natranaerovirga pectinivora* | 0.000 | 0.006 | 0.000 |
|  | *Oscillibacter* | *Oscillibacter ruminantium* | 0.000 | 0.683 | 0.000 |
|  | *Oscillibacter* | *Oscillibacter valericigenes* | 0.000 | 0.149 | 0.000 |
|  | *Paenibacillus* | *Paenibacillus etheri* | 0.126 | 0.000 | 0.000 |
|  | *Peptococcus* | *Peptococcus niger* | 0.000 | 0.006 | 0.000 |
|  | *Peptoniphilus* | *Peptoniphilus grossensis* | 0.000 | 0.096 | 0.000 |
|  | *Peptoniphilus* | *Peptoniphilus obesi* | 0.000 | 0.006 | 0.000 |
|  | *Peptostreptococcus* | *Peptostreptococcus anaerobius* | 0.000 | 0.066 | 0.000 |
|  | *Phascolarctobacterium* | *Phascolarctobacterium faecium* | 0.000 | 0.154 | 0.000 |
|  | *Phascolarctobacterium* | *Phascolarctobacterium succinatutens* | 0.000 | 0.186 | 0.000 |
|  | *Robinsoniella* | *Robinsoniella peoriensis* | 0.000 | 0.006 | 0.000 |
|  | *Romboutsia* | *Romboutsia timonensis* | 0.010 | 0.879 | 0.000 |
|  | *Roseburia* | *Roseburia faecis* | 0.000 | 0.006 | 0.000 |
|  | *Roseburia* | *Roseburia inulinivorans* | 0.000 | 0.474 | 0.000 |
|  | *Ruminiclostridium* | *Ruminiclostridium thermocellum* | 0.001 | 0.109 | 0.000 |
|  | *Ruminococcus* | *Ruminococcus bromii* | 0.000 | 0.295 | 0.000 |
|  | *Ruminococcus* | *Ruminococcus champanellensis* | 0.000 | 0.256 | 0.000 |
|  | *Ruminococcus* | *Ruminococcus faecis* | 0.000 | 0.021 | 0.000 |
|  | *Blautia* | *Ruminococcus gnavus* | 0.000 | 0.032 | 0.000 |
|  | *Saccharofermentans* | *Saccharofermentans acetigenes* | 0.000 | 0.009 | 0.000 |
|  | *Selenomonas* | *Selenomonas bovis* | 0.000 | 0.026 | 0.000 |
|  | *Solobacterium* | *Solobacterium moorei* | 0.000 | 0.190 | 0.000 |
|  | *Sporobacter* | *Sporobacter termitidis* | 0.000 | 0.222 | 0.000 |
|  | *Sporolactobacillus* | *Sporolactobacillus nakayamae* | 0.038 | 0.000 | 0.000 |
|  | *Staphylococcus* | *Staphylococcus cohnii* | 0.000 | 0.000 | 0.010 |
|  | *Staphylococcus* | *Staphylococcus sciuri* | 0.000 | 0.000 | 0.044 |
|  | *Staphylococcus* | *Staphylococcus xylosus* | 0.006 | 0.000 | 1.245 |
|  | *Streptococcus* | *Streptococcus pasteurianus* | 0.000 | 0.092 | 0.000 |
|  | *Succiniclasticum* | *Succiniclasticum ruminis* | 0.000 | 0.017 | 0.000 |
|  | *Tetragenococcus* | *Tetragenococcus halophilus* | 0.000 | 0.000 | 0.919 |
|  | *Tissierella* | *Tissierella praeacuta* | 0.000 | 0.006 | 0.000 |
|  | *Turicibacter* | *Turicibacter sanguinis* | 0.000 | 0.224 | 0.000 |
|  | *Vallitalea* | *Vallitalea pronyensis* | 0.000 | 0.049 | 0.000 |
|  | *Veillonella* | *Veillonella dispar* | 0.000 | 0.009 | 0.000 |
| Fusobacteria | *Fusobacterium* | *Fusobacterium nucleatum* | 0.000 | 0.043 | 0.000 |
|  | *Fusobacterium* | *Fusobacterium varium* | 0.000 | 0.036 | 0.000 |
| Proteobacteria | *Actinobacillus* | *Actinobacillus porcinus* | 0.000 | 0.049 | 0.000 |
|  | *Azonexus* | *Azonexus caeni* | 0.000 | 0.006 | 0.000 |
|  | *Desulfovibrio* | *Desulfovibrio desulfuricans* | 0.000 | 0.032 | 0.000 |
|  | *Desulfovibrio* | *Desulfovibrio simplex* | 0.000 | 0.047 | 0.000 |
|  | *Diaphorobacter* | *Diaphorobacter polyhydroxybutyrativorans* | 0.000 | 0.006 | 0.000 |
|  | *Duodenibacillus* | *Duodenibacillus massiliensis* | 0.001 | 0.083 | 0.000 |
|  | *Erwinia* | *Erwinia aphidicola* | 0.037 | 0.017 | 2.773 |
|  | *Erwinia* | *Erwinia typographi* | 0.000 | 0.000 | 0.010 |
|  | *Escherichia* | *Escherichia fergusonii* | 0.010 | 0.946 | 0.000 |
|  | *Geobacter* | *Geobacter pelophilus* | 0.000 | 0.006 | 0.000 |
|  | *Lacibacterium* | *Lacibacterium aquatile* | 0.000 | 0.006 | 0.000 |
|  | *Obesumbacterium* | *Obesumbacterium proteus* | 0.000 | 0.000 | 0.010 |
|  | *Oligella* | *Oligella ureolytica* | 0.000 | 0.006 | 0.000 |
|  | *Ottowia* | *Ottowia shaoguanensis* | 0.000 | 0.006 | 0.000 |
|  | *Pantoea* | *Pantoea conspicua* | 0.010 | 0.000 | 0.000 |
|  | *Pantoea* | *Pantoea deleyi* | 0.000 | 0.000 | 3.117 |
|  | *Parasutterella* | *Parasutterella excrementihominis* | 0.001 | 0.032 | 0.000 |
|  | *Pelobacter* | *Pelobacter propionicus* | 0.000 | 0.006 | 0.000 |
|  | *Povalibacter* | *Povalibacter uvarum* | 0.000 | 0.094 | 0.000 |
|  | *Pseudolabrys* | *Pseudolabrys taiwanensis* | 0.000 | 0.006 | 0.000 |
|  | *Pseudomonas* | *Pseudomonas gessardii* | 0.188 | 0.000 | 0.000 |
|  | *Pseudomonas* | *Pseudomonas trivialis* | 0.000 | 0.000 | 1.116 |
|  | *Pseudomonas* | *Pseudomonas lactis* | 0.013 | 0.000 | 0.000 |
|  | *Pseudomonas* | *Pseudomonas weihenstephanensis* | 0.000 | 0.000 | 5.285 |
|  | *Racemicystis* | *Racemicystis persica* | 0.000 | 0.015 | 0.000 |
|  | *Rahnella* | *Rahnella genomosp.* | 0.013 | 0.000 | 0.000 |
|  | *Rahnella* | *Rahnella victoriana* | 0.000 | 0.000 | 0.272 |
|  | *Rhodovibrio* | *Rhodovibrio sodomensis* | 0.000 | 0.038 | 0.000 |
|  | *Rubellimicrobium* | *Rubellimicrobium roseum* | 0.000 | 0.006 | 0.000 |
|  | *Smithella* | *Smithella propionica* | 0.000 | 0.013 | 0.000 |
|  | *Snodgrassella* | *Snodgrassella alvi* | 0.000 | 0.006 | 0.000 |
|  | *Sutterella* | *Sutterella massiliensis* | 0.000 | 0.194 | 0.000 |
|  | *Sutterella* | *Sutterella wadsworthensis* | 0.001 | 0.809 | 0.000 |
|  | *Syntrophus* | *Syntrophus aciditrophicus* | 0.000 | 0.006 | 0.000 |
|  | *Vulgatibacter* | *Vulgatibacter incomptus* | 0.000 | 0.009 | 0.000 |
|  | *Wenzhouxiangella* | *Wenzhouxiangella marina* | 0.000 | 0.006 | 0.000 |
| Spirochaetes | *Treponema* | *Treponema berlinense* | 0.000 | 0.006 | 0.000 |
|  | *Treponema* | *Treponema bryantii* | 0.000 | 0.006 | 0.000 |
|  | *Treponema* | *Treponema rectale* | 0.000 | 0.006 | 0.000 |
| Tenericutes | *Acholeplasma* | *Acholeplasma parvum* | 0.000 | 0.006 | 0.000 |
| Verrucomicrobia | *Akkermansia* | *Akkermansia muciniphila* | 0.000 | 0.083 | 0.000 |
